# Experimentally inducing dreams of remote emotional memory

**DOI:** 10.1093/sleepadvances/zpaf054

**Published:** 2025-08-18

**Authors:** Chadwick C Frost, Erin J Wamsley

**Affiliations:** Department of Psychology & Program in Neuroscience, Furman University, Greenville, SC, USA; Department of Psychology & Program in Neuroscience, Furman University, Greenville, SC, USA

**Keywords:** memory reactivation, memory reconsolidation, dream content, dream affect, sleep, EEG

## Abstract

People frequently dream of recent experiences, which may reflect the consolidation of memories in the sleeping brain. Many studies demonstrate that experimentally introducing new learning prior to sleep can induce dreams of recently encoded memories. Here, for the first time, we tested whether activating a *remote* memory just before sleep can similarly induce participants to dream about the remote past. Participants (*N* = 34) completed an Autobiographical Emotional Memory Task (AEMT), in which they recalled and wrote about an emotionally negative remote memory prior to a daytime nap. In a control condition, participants instead wrote about designing a new college course. As hypothesized, the AEMT induced emotionally negative dreams related to the remote memory activated before sleep. Specifically, according to judge ratings, dreams incorporated content from the remote memory recalled during the AEMT to a greater degree than they incorporated content from the control task. While participants themselves did not perceive their dreams as more strongly related to the AEMT memory than the control task, they did rate their dreams as more emotionally negative following the AEMT. This shows it is possible to experimentally induce dreams of a specific remote memory by activating it before sleep. These findings are discussed in light of the hypothesis that dream content might be influenced by the reconsolidation of recently reactivated remote memory.

**Statement of Significance**

This research is among the first to experimentally study how dreams incorporate remote memories from the long past. This is important because sleep may function not only to stabilize recent memories, but also to update older memories with newly learned information. Studying the appearance of remote memories in dreams may provide a window into how the process unfolds in the sleeping brain.

## Introduction

While sleep is hypothesized to have a number of different functions, recent research has especially highlighted its role in strengthening and stabilizing new memories [1–3]. Intriguingly, dream content appears to reflect this process of memory consolidation. Research participants frequently dream about learning experiences encountered before sleep, and when they do, memory for those experiences is improved [4]. But besides consolidating recently formed memory, emerging evidence suggests sleep may also play a role in reconsolidating *remote* memory networks that have been reactivated and updated during wake [5]. In the current study, we attempted to induce dreams of a remote autobiographical memory by reactivating it just before sleep, with the goal of better understanding how and why remote memories are incorporated into dreams.

In laboratory studies, presleep learning experiences are often incorporated into the content of subsequent dreams [6–12], and dreaming about learning tasks predicts post-sleep improvements in performance [4, 6, 10, 13–15]. For example, in a series of studies, participants completed a virtual maze navigation task before a night of sleep. Those who recalled dreams about the maze showed greater improvement on the task after sleep, compared to those who did not [15, 16]. In this work, “dreaming” is defined as any subjective mental experience during sleep, regardless of sleep stage. A 2023 meta-analysis of 16 studies of this kind established that participants who dream about presleep learning tasks reliably outperform those who do not [4]. While correlational, this evidence suggests that the reactivation and consolidation of recent learning experiences during sleep can be observed in dream content.

Sleep is also thought to play a role in memory *reconsolidation*, which takes place when an already-consolidated memory is reactivated and temporarily destabilized [5, 17–19], enabling it to be updated with new information. For example, reminding participants of a remote memory just before learning new, related information alters the remote memory representation by incorporating material from the new learning experience [20, 21]. Sleep may contribute to this process by restabilizing remote memories after they have been reactivated and destabilized during wakefulness [5, 22].

Potentially, dreams might reflect the reconsolidation of remote memories during sleep. People frequently dream about memories from the remote past, especially when they have just encountered a related experience that reminds them of one. In retrospective studies, participants typically identify >50% of their dreams as originating from at least one specific past experience of theirs [23–25]. Most often, these memory sources are from the recent past. However, it is also common for participants to identify remote memories as contributors to a dream [23–25]. In one recent study, 30% of dreams incorporated a memory from the remote past [24], in some cases alongside a semantically related recent memory [24, 25]. As one illustrative example of this type of dream, a participant reported:

“I was in a grocery store and it was kind of like sleep study subjects which was a little funny. Because everyone was hooked up to the wiring and stuff and we all had matching pajamas…” [24]

This dream is most obviously related to the participant’s current experience of being in a sleep study, but they also attributed it to a book series they had read as a child, where children *wearing matching pajamas* were being tested, hooked up to wiring [24]. Presleep experiences have similarly induced dreams of semantically related remote memory in other studies. For example, playing a skiing-themed arcade game triggered participants to dream of remote past skiing experience [12], and playing the game *Tetris* led participants to dream about remote memories of that game [11]. Dreams that intermingle details of a recent experience with related memories of the remote past could potentially reflect the process of memory reconsolidation. Hypothetically, a presleep experience could serve as a reminder cue that reactivates and destabilizes a semantically related remote memory, making it amenable to alternation. During subsequent sleep, the remote memory is then restabilized via a neural process that influences the ongoing conscious content of dreams.

Our current understanding of how presleep experience might trigger dreams of remote memory is limited. Evidence that this occurs comes exclusively from retrospective studies, in which participants report dreams and then are subsequently asked to identify past memory sources that they believe contributed to the content of those dreams. While useful, these retrospective data are subject to bias. In identifying the memory sources of their dreams, participants may incorrectly perceive dream content to be caused by a particular past episode, when in fact the similarity between a dream and a remote memory is due to chance coincidence. Determining whether a particular remote memory actually *caused* dream content requires an experimental manipulation that targets that particular remote memory for reactivation.

In the current study, we experimentally reactivated a remote memory by having participants intentionally recall an emotionally negative memory just before a daytime nap. We targeted emotionally negative memory based on evidence that negative memories are preferentially reactivated and consolidated during sleep [26]. For example, after viewing a series of scenes containing negative and neutral objects, sleep leads to enhanced memory for negative objects, but not for neutral objects or their backgrounds [27, 28]. In one study using this paradigm, memory for negative objects selectively benefitted from sleep only in participants who recalled dreaming [29]. More generally, there is evidence that dream content preferentially reflects emotionally negative memory [30] (though the extent to which this is true depends on the method by which dream affect is measured [31]), and that emotional experiences are more likely to be incorporated into dreaming than neutral experiences [32]. Thus, in the current study, we reasoned that remote memories with negative valence might have a strong likelihood of affecting dream content. We hypothesized that activating a remote emotional memory before sleep would induce dream content related to that memory and shift dream affect to be more emotionally negative.

## Materials and Methods

### Participants

To be eligible for the study, participants were required to be college students between 18 and 30 years old and fluent in English. To minimize the potential for adverse effects resulting from the emotional memory task used in this study, participants were ineligible to enroll if they reported a diagnosis of post-traumatic stress disorder (PTSD). If determined as eligible, participants could sign up for two study sessions with start times between 11:00 am and 5:00 pm The final sample was *N* = 34 undergraduate students at Furman University, 18–22 years old, who reported never having been diagnosed with PTSD (mean age = 19.65±1.32 SD; 13 males/21 females). Participants were compensated $10/h or received credit in an introductory psychology course. EEG (electroencephalography) from the experimental condition was unusable for 2 participants, due to excessive artifact (*N* = 1) or other technical difficulty (*N* = 1). These two participants are excluded from analyses that require EEG data, but are included in analyses of behavioral data.

### Procedure

**Figure 1:**
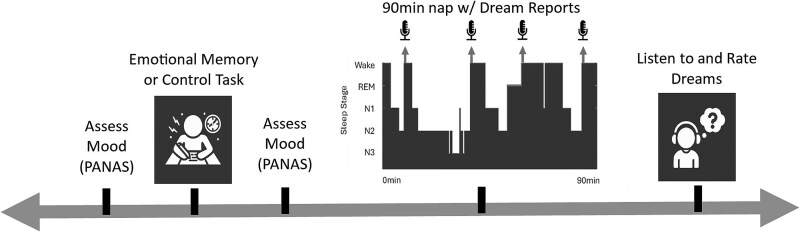
Experimental timeline. After a baseline mood assessment, participants completed either the AEMT (emotional memory recall task) or a control task. Mood was measured again just prior to a 90-min nap, during which participants reported up to 4 dreams (microphone icons indicate report collection timepoints relative to sleep architecture in one example participant). PANAS, positive and negative affect scale.

Upon arrival for their first laboratory visit, participants signed informed consent and completed a survey about sleep habits and demographic information, as well as the Stanford Sleepiness Scale, a standardized measure of state sleepiness [33]. Participants were then prepared for EEG recording before completing either the Autobiographical Emotional Memory Task (AEMT) or a control task, as described below. Experimental condition (AEMT vs. control task) was a within-subjects variable, with each participant completing both conditions during separate laboratory visits, in counterbalanced order. Participants also completed the Positive and Negative Affect Scale (PANAS) [34] just before and after the AEMT or control task (Figure 1).

Participants then lay down to begin a 90-min nap opportunity, during which they provided up to four dream reports. For each report, participants were awoken by calling their name over an intercom and prompting them to “please report everything that was just going through your mind.” The first report was collected just following sleep onset, 5 min after participants first entered stage N1 sleep. After returning to sleep, up to 2 additional reports were collected, each following at least 15 continuous minutes of sleep. Finally, at the end of the 90 min, one additional report was collected regardless of the stage of sleep or wakefulness. This specific protocol for awakening participants to report dreams was chosen in order to sample the largest amount of content possible during a short nap, while ensuring that dream reports from multiple sleep stages would be represented. This awakening protocol is similar to those that we have employed in numerous past investigations (eg, [12, 15]).

Following the nap, participants listened to the audio recording of each of their reports and used a 7-point scale to rate the extent to which each related to the autobiographical memory they wrote about before sleep (in the AEMT condition), or the topic of the control writing task (in the control condition). A 7-point Likert scale for describing incorporation was chosen as a simple method of assessing this construct, given that no existing scales for this specific purpose exist. Scale anchors were 1 = the dream is not related to the presleep writing response and 7 = the dream is strongly related to the presleep writing response. Full instructions provided to judges are available along with other study material, data, and analysis code on Open Science Framework at https://osf.io/ewmp7. Participants also rated the emotional valence of each dream on a 7-point scale and indicated whether the dream was related to any other episodic memories from their personal past. If participants indicated the report was related to another autobiographical memory, they described the memory, rated its emotional valence, and indicated when it occurred (past day/past week/past month/past year/>1 year ago).

### Judge scoring of dream reports

Following data collection, audio recordings were transcribed and rated by two independent judges, blind to experimental condition. For each participant, judges were provided with all dream reports from that participant in randomized order, from both experimental conditions, alongside the participant’s written responses from both experimental conditions. For each report, judges first determined whether the report contained a description of any mental content (as opposed to a lack of recall, such as “I don’t know”/“I don’t remember”/“there was nothing”). They then assessed the extent to which the report incorporated content related to the AEMT writing response (1–7 scale) and the extent to which it incorporated content related to the control condition writing response (1–7 scale). Inter-rater agreement for the presence of content was near-perfect (kappa = .94) and for memory incorporation was substantial (weighted kappa = .66) [35]. We tested for incorporation of the AEMT memory into dream content by examining whether dreams following the AEMT were judged as more similar to participants’ written AEMT response than to the control task writing response.

### E‌EG recording

EEG (electroencephalography) was recorded from the F3, F4, C3, C4, O1, and O2 scalp locations, with electrodes placed according to the international 10–20 system of electrode placement. For the purposes of determining sleep stage, electromyography was recorded from the chin, and eye movements were recorded using electrodes placed on the left and right outer canthus. Data were recorded at 500 Hz using a BrainAmp amplifier (Brain Products, www.brainproducts.com).

### Sleep staging

Following data collection, sleep stage was scored offline according to the standards set by the American Academy of Sleep Medicine [36]. For purposes of analysis, each verbal dream report was categorized according to the last sleep stage that participants were in prior to awakening and providing the report. For 18 reports, the sleep stage prior to awakening could not be accurately determined, due to either excessive artifact in the EEG or due to a failure to accurately record the moment that participants were awakened. Furthermore, because the final report was collected irrespective of sleep/wake stage, and due to imprecision inherent to the process of conducting awakenings earlier in the nap, some reports were collected while the participant was awake. Reports collected from wakefulness are excluded from all analyses except those specifically testing the effects of sleep/wake state.

### AEMT and control tasks

The AEMT is a writing task that prompts participants to recall a highly emotional remote autobiographical memory, and has been shown to be effective in experimentally inducing specific emotions [37]. In the AEMT condition, participants responded to a prompt stating: “Please describe in detail the one situation that has made you the most angry you have been in your life. Describe it in such a way that the person reading the description would become angry just from reading it.” Anger was chosen as the prompt emotion because previous research determined that writing about anger induced the largest change in emotion from before to after the writing task [37]. Our goal was to choose an emotion that would produce the most engagement and salience during the writing task. Participants in the control condition instead wrote about designing their ideal college course, responding to a prompt stating: “Please describe in detail a new course you would design for your college. Include the subject, learning objectives, and teaching methods you would implement.” In both conditions, participants were given 5 min to respond to the prompt. They were instructed to try to use the full 5 min, and were not able to advance to the next screen of the survey until the 5 min was up. In the experimental condition, participants also reported when the emotional memory had occurred.

### Analysis methods

Statistical analyses were conducted in R [38]. The effects of experimental condition on dreaming were tested using hierarchical linear models, which enabled us to account for the fact that each participant provided multiple different dream reports across the course of the nap. Specifically, we utilized random-intercept mixed-effect models, with observations grouped by the random factor of subject. Mixed-effect models were conducted using the lme4 and lmerTest packages for R [39]. Statistical significance was assessed using Wald chi-squared tests, with Satterthwaite’s method of calculating the degrees of freedom. Subsequent pairwise comparisons were then conducted on the estimated marginal means using the emmeans package in R [40]. Only analyses including sleep/wake stage as a factor include wake reports. From all other analyses, reports collected from wakefulness are excluded.

Ratings of the extent to which dream reports related to the presleep writing prompt were not normally distributed (right-skewed), and so were log-transformed prior to analysis. However, even after log-transformation moderate skewness remained. As a robustness check, we therefore also tested the effect of experimental condition on relatedness ratings using cumulative link mixed models (using the ordinal package for R [41]), which do not assume a normal distribution of values. These latter analyses yielded the same results as the primary statistical approach, and can be viewed in our posted analysis script on Open Science Framework (https://osf.io/ewmp7/).

Paired samples *t*-tests were used to determine the effect of experimental condition on dream emotion, presleep mood, incorporation of other autobiographical memories, and emotion ratings of those other autobiographical memories.

## Results

A total of 224 reports were collected, including 112 in the control condition and 112 in the AEMT condition. Each participant contributed an average of 3.32 ± 0.66 SD reports (range: 2–5). Participants recalled mental content in 78.57% (*N* = 176) of reports, which was similar between conditions (*N* = 87 content-filled reports in the control condition and *N* = 89 in the AEMT condition). Participants slept for an average of 46.54 ± 21.96 SD min during the nap, and sleep stage composition was similar between conditions, as illustrated in Table 1. Table 2 reports the proportion of dream reports collected from each stage of sleep.

**Table 1:** Sleep Architecture by Experimental Condition

	Experimental condition			
Sleep stage	AEMT		Control	
N1 min	10.37	± 5.45	10.89	± 7.36
N2 min	29.18	± 15.99	29.52	± 16.36
N3 min*	6.55	± 4.39	7.52	± 4.25
REM min*	5.80	± 3.27	8.22	± 6.68
Total Sleep Time (min)	46.37	± 22.70	46.72	± 21.50
% participants obtaining N3 sleep		69% (*n* = 22)		62% (*n* = 21)
% participants obtaining REM sleep		31% (*n* = 10)		26% (*n* = 9)

Mean number of minutes ± SD. REM = Rapid Eye Movement.

^*^Among those who spent any time in this sleep stage.

**Table 2:** Number of Dream Reports by Sleep Stage

Sleep stage	*N* without content	*N* with content	% with content	% from stage
Wake*	12	84	87.50	21.43
N1	10	60	85.71	15.63
N2	44	134	75.28	39.73
N3	22	44	66.67	14.73
REM	4	16	80.00	4.46

A report was considered to have content when the participant recalled any mental experience. REM = Rapid Eye Movement. % with content = percent of all awakenings from this stage that yielded some mental content. % from sleep stage = percent of all awakenings from this sleep stage. For an additional *N* = 18 reports, sleep stage could not be accurately determined.

^*^Reports collected during wakefulness are excluded from all analyses except those specifically testing the effect of sleep/wake state on the outcome measures.

### Effect of the AEMT on presleep mood

**Figure 2:**
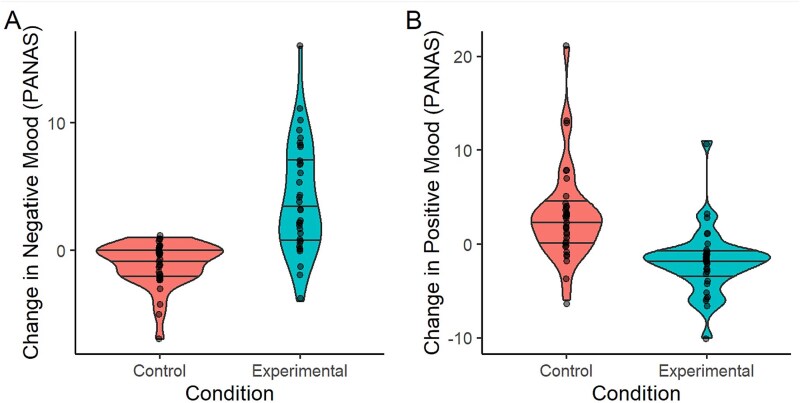
Effect of AEMT on mood. (A) Effect of experimental condition on positive affect. (B) Effect of experimental condition on negative affect. PANAS, positive and negative affect scale. Mood change is calculated as post-task PANAS − Pre-task PANAS. The width of the violin plot represents the probability density. Horizontal lines represent the 25th, 50th, and 75th percentiles.

The AEMT strongly affected presleep mood (Figure 2). Specifically, PANAS negative scores increased significantly more following the AEMT than the control task (*t*(33) = 6.97, *p* < .001, *d* = 1.19; Figure 2, A), while PANAS positive scores decreased significantly more following the AEMT than the control task (*t*(59) = 4.69, *p* < .001, *d* = 1.08; Figure 2, B).

### Effect of the AEMT on dream content

#### Judge ratings

**Figure 3:**
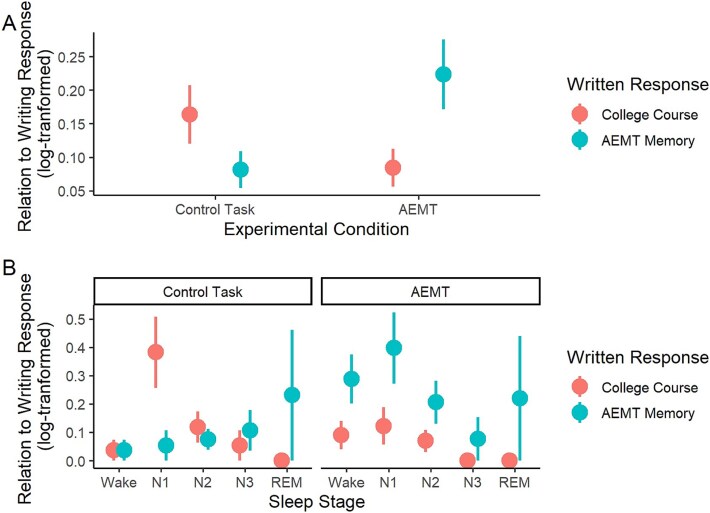
Effect of AEMT on dream content. According to judge ratings. (A) Dreams in the AEMT condition were more strongly related to the AEMT memory than to the control task writing response. (B) Dream content reflected the reactivated AEMT memory especially during N1, N2, and wake. AEMT, autobiographical emotional memory task; REM, rapid eye movement. Error bars = SEM.

Participants dreamed about the memory recalled during the AEMT more than they dreamed about the control task, according to judge ratings. Specifically, in the AEMT condition, dreams resembled the AEMT writing response significantly more than the control writing response (*t*(226) = 2.53, *p* = .012, *d* = 0.44). Conversely, in the control condition, dreams were no more similar to the control writing response than they were to the AEMT response (*t*(226) = 1.52, *p* = .130; report condition × response condition interaction: Wald *χ*^2^(4) = 8.24, *p* = .004, $\beta =0.22$; Figure 3, A). There was no effect of awakening number on judge ratings (Wald *χ*^2^ (4) = 2.63, *p*=.62).

In a model including sleep/wake stage, the effect of the AEMT on dream content was marginally dependent on sleep/wake stage (3-way stage × report condition × response condition interaction: Wald *χ*^2^ (4) = 7.97, *p* = .093; Figure 3, B). Pairwise comparisons revealed that in the experimental condition, reports were more similar to the AEMT response than the control writing response selectively in wake (*t*(311) = 2.68, *p* = .008, *d* = 0.85) and N1 (*t*(317) = 3.06, *p* = .002, *d* = 1.14), with marginal significance in N2 (*t*(302) = 1.72, *p* = .087, *d* = 0.42; Figure 3, B). In the control condition, dreams were more strongly related to the control than the AEMT prompt only in N1 (*t*(317) = 2.37, *p* = .002, *d* = 0.88; Figure 3B).

#### Participant ratings

**Figure 4:**
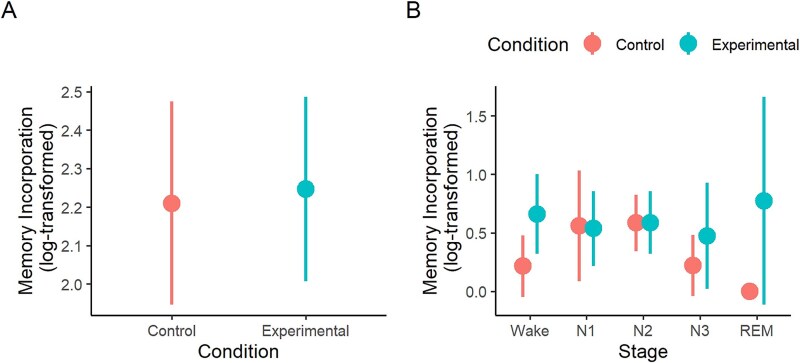
Effect of AEMT on dream content according to participant ratings. (A) Participants did not perceive dreams in the AEMT condition to be more strongly related to the presleep task than in the control condition. (B) Effect of AEMT on dream content by sleep stage. AEMT, autobiographical emotional memory task; REM, rapid eye movement. Error bars = SEM.

**Figure 5:**
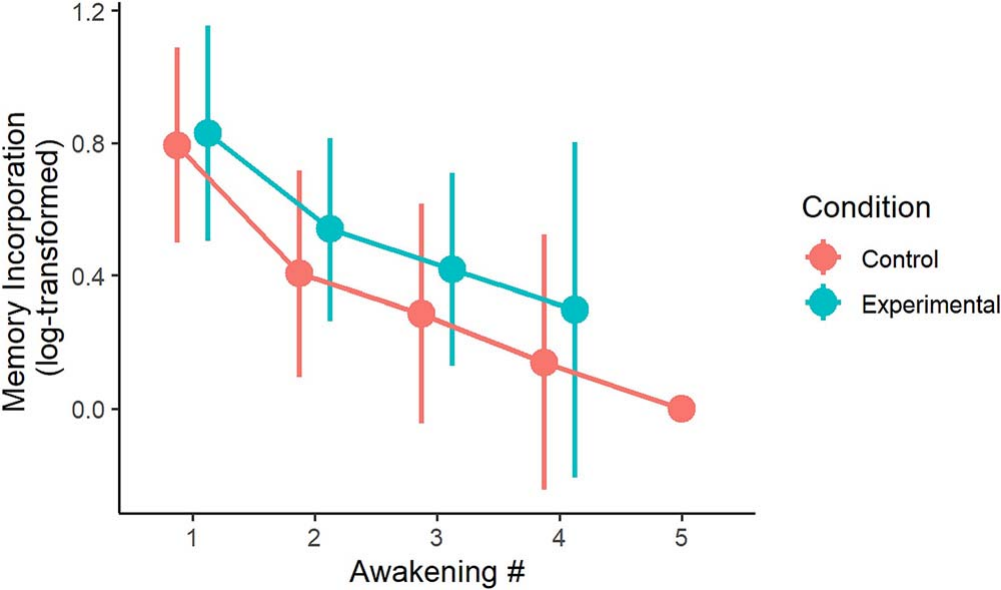
Participant ratings of presleep task incorporation decline across successive awakenings. Error bars = SEM.

In contrast to judge ratings, participants did not perceive their own dreams as more strongly influenced by the AEMT than the control task (nonsignificant main effect of condition: Wald *χ*^2^ (1) = 0.03, *p* = .858; Figure 4, A). Instead, participants perceived above-zero similarity between their dreams and the presleep task in both conditions (AEMT mean relatedness rating = 2.2, SD = 1.22, 95% CI = 1.61% to 2.34%; Control mean relatedness rating = 2.22, SD = 1.45, 95% CI = 1.46% to 2.29%). Across both conditions, there was a trend for participants to view their dreams as more strongly related to the presleep task during the first report collected, with this effect declining across time (main effect of awakening number: Wald *χ*^2^ (4) = 9.23, *p* = .056, Figure 5).

A model examining sleep/wake stage revealed that participants perceived their waking thoughts to incorporate the reactivated memory, but not their dreams. Neither the main effect of sleep stage (Wald *χ*^2^ (4) = 6.86, *p* = .144), nor its interaction with condition was significant (Wald *χ*^2^ (4) = 4.97, *p* = .290). While the effect of experimental condition became statistically significant in this model (Wald *χ*^2^ (1) = 4.75, *p* = .029, *d* = 0.43), this was driven by the inclusion of reports collected from wake (Figure 4, B). Participants perceived their waking experience to be more influenced by the AEMT than the control task (*t*(226) = 2.18, *p* = .031, *d* = 0.68), but this effect did not near significance for any sleep stage (Figure 4, B).

#### Examples of memory incorporation

As anticipated, dreams following the AEMT were never an exact replay of the reactivated memory. On a 1–7 scale, where a “7” would be a full replication of the recalled remote memory, the maximum rating assigned to any dream was a “5.” Instead of replaying the memory in its entirety, dreams commonly incorporate isolated elements of the reactivated memory. For example, one participant reported the following childhood memory:

“… One day, I came home from school and found my Holiday Barbie, my most prized possession, decapitated. My brother, who was four at the time had gotten a hold of her while I was gone and ripped off her head.”

In a subsequent dream, this participant reported they were “at home with my family and… with my little brother.” A different participant, after reporting a memory of a breakup with a girlfriend, dreamed “I was listening to music… the song that was playing was my girlfriend’s favorite song at the time we were breaking up.” Other examples were of a similar nature, in which the dream was only loosely related to the experience, containing one or more elements from the recalled memory, but in a different overall context than the source memory.

### Effect of the AEMT on dream emotion

**Figure 6:**
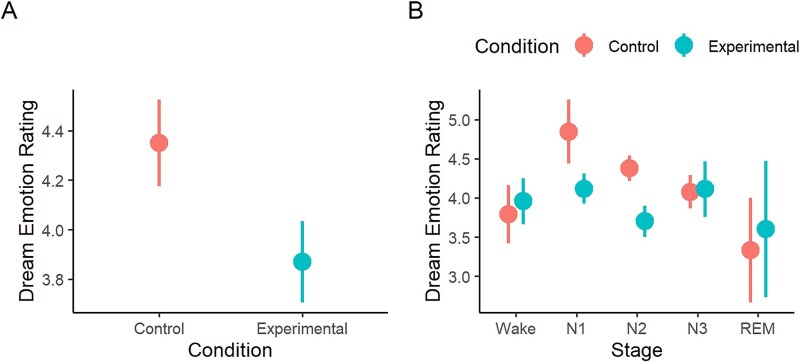
Participant ratings of dream emotion. (A) Dreams in the AEMT condition were more emotionally negative than dreams in the control condition. (B) Dream emotion was more negative in the AEMT than the control condition specifically in stages N1 and N2 sleep. AEMT, autobiographical emotional memory task; REM, rapid eye movement. Error bars = SEM.

As hypothesized, participants rated their dreams as more emotionally negative after the AEMT than after the control task (experimental condition: M = 3.84, SD = 0.84; control condition: M = 4.37, SD = 0.96; Wald *χ*^2^ (1) = 8.32, *p* = .004, *β* = 0.22, Figure 6, A). Dream affect did not vary by awakening number (Wald *χ*^2^ (4) = 1.31, *p* = .859), and there was no interaction between condition and awakening number (Wald *χ*^2^ (3) = 2.96, *p* = .398).

In a model examining the effect of sleep/wake stage, the AEMT significantly impacted dream affect only in stage N2 (*t*(149) = 2.29, *p* = .024, *d* = 0.57), with marginal significance in stage N1 (*t*(159) = 1.71, *p* = .089, *d* = 0.64; Figure 6, B). However, this may merely be a consequence of the larger number of reports collected from N2, as the condition × sleep stage interaction effect was not significant (Wald *χ*^2^ (4) = 5.21, *p* = .266). The AEMT did not affect the emotion of wake reports (*t*(155) = 0.47, *p* = .636, *d* = 0.15).

### Other past episodic memories incorporated into dreams

**Figure 7:**
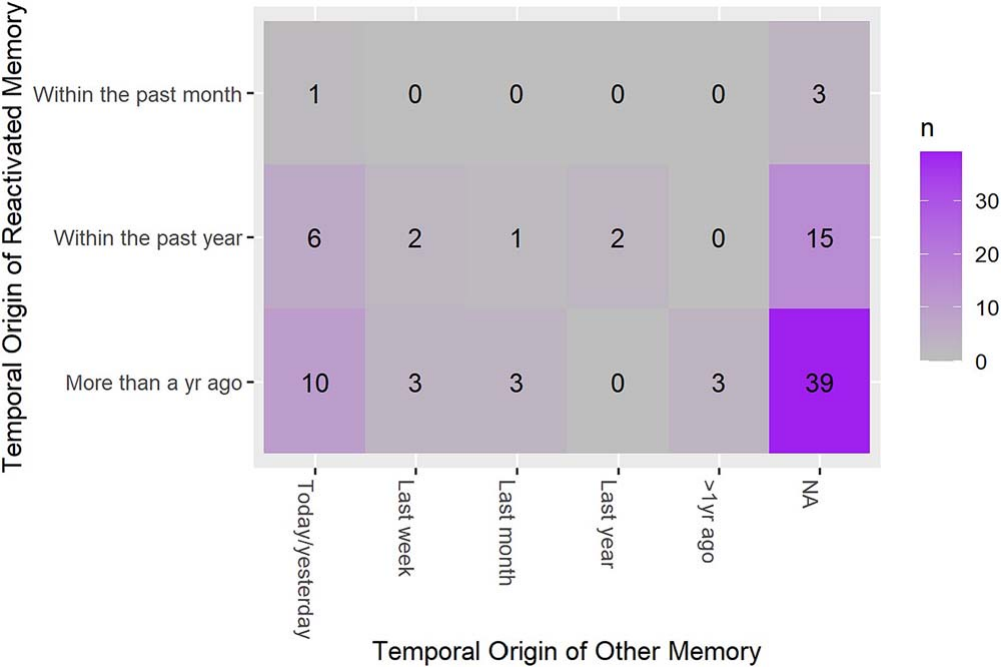
Temporal origin of dream memory sources in the AEMT condition. Frequency counts represent the number of dreams, classified both by the temporal origin of the remote memory reactivated during the AEMT (*y*-axis) and the temporal origin of other past episodes identified as a source for the same dream (*x*-axis). NA = dreams for which participants did not identify another past episodic memory source. These data represent dreams from the experimental (AEMT) condition only. AEMT, autobiographical emotional memory task.

Participants indicated that 63 dreams (35.8% of all content-filled dreams) also related to another past episodic memory. Most commonly, these were memories from the recent past (Figure 7). These other episodic memory sources were also more emotionally negative in dreams following the AEMT, compared to dreams following the control task (Wald *χ*^2^ (1) = 5.61, *p* = .018, $\beta$ = −0.94).

## Discussion

Recalling a remote emotional memory before sleep influenced both the content and emotional tone of participants’ dreams. This is the first experimental demonstration that dreams of remote memory can be induced via a presleep manipulation. Together with other work, these observations suggest that in our daily lives, long-past experiences may become reactivated and incorporated into dreams when we are reminded of them prior to sleep. However, remote memory fragments also appear in dreams even when there has been no apparent reactivation of those memories prior to sleep [25]. Although this preliminary study did not measure changes in the memory trace following sleep and dreaming, we speculate that the appearance of remote memory fragments in dreams could be related to sleep’s role in memory *reconsolidation*.

### Relation to prior work on dreaming and memory

The current study is the first to experimentally induce dreams of a remote memory, but it has long been known that dreams include content drawn from the remote past [23, 42]. Is it possible that all dreams of remote memory are triggered by some covert reactivation of a past experience prior to sleep? While this cannot be ruled out, more likely, presleep reactivation increases the likelihood that a remote past memory will be incorporated in dream content, without being strictly required for incorporation to occur.

This influence of presleep reactivation on a dream appears to be strongest early in the sleep phase, as dreams were most strongly related to the AEMT memory early in the nap, with relatedness declining in later awakenings. This echoes prior studies that similarly observed incorporation of a presleep learning task to be strongest during early night awakenings, and then decline with time [25, 43]. Notably, this mirrors an analogous temporal gradient in the strength of neural-level memory reactivation, which is also generally strongest in early sleep, immediately following the target experience [44, 45].

The autobiographical memory task significantly affected dreaming only in NREM sleep stages N1 and N2. Since the 1960s, researchers have debated whether dreaming during NREM sleep, and especially during early, light NREM stages, can be considered part of the same phenomenon as the longer, better-remembered dreams of rapid eye movement (REM) sleep. The most robust difference between subjective experience recalled from early NREM sleep and later stages is that dreams in early NREM tend to be shorter than, for example, late-night REM sleep dreams [46–48]. Very early in N1, dreams also can be more “thought-like,” but these give way to hallucinatory experiences within a short timeframe [46, 49]. There is little empirical evidence that dreaming is categorically, qualitatively different in early NREM, above and beyond the well-described difference in dream length. To the contrary, highly vivid, emotional, and complex dreams can occur in any sleep stage, especially during periods of high circadian-driven cortical activation, such as a daytime nap [48]. Thus, while we cannot say that the effects we report here would generalize to REM sleep, we do conclude that our experimental manipulation affected “dreaming.”

Intriguingly, the autobiographical memory task affected not only dream reports but also reports of waking thought. This is consistent with recent research from our group and others demonstrating that memory reactivation and consolidation occur during resting wakefulness, a manner similar to how these processes unfold during sleep (for a review, see [50]). That reactivating an autobiographical memory prior to sleep affected both dreaming and waking thought could indicate that this triggered consolidation-related processes in both states. However, that emotion was affected only during sleep could indicate an affective component of this processing that is sleep-specific.

In line with prior research, dreams following the AEMT were never an exact reiteration of the memory recalled before sleep. Instead, dreams incorporated isolated elements of that memory, unbound from their original episodic context and intermingled with details drawn from other past episodes. This is a well-known feature of dreams of recent experience, which rarely incorporate more than a few fragments of an episodic memory [51, 52], and routinely combine details drawn from multiple different past episodes [25]. In the current study, other past episodic sources of participants’ dreams were also more negatively valanced following the AEMT, in comparison to the control condition. This suggests the possibility that the sleeping brain may activate and combine memory traces that are affectively congruent.

### Judge vs. participant ratings of dream incorporation

The effect of experimental condition on dream incorporation was detected in judge ratings, but not in participants’ own ratings. As mentioned previously, participants perceived their dreams as related to the writing task in both conditions. This could be explained by the fact that participants have more information about their own memories and dreams than present in the written dream report. For example, if a feature of a remote memory that was not described in participants’ written response to the AEMT appears in a subsequent dream, the participant may conclude that the dream is related, even though an outside observer would not be able detect this. Alternatively, the difference between judge and participant ratings of incorporation might be explained by demand characteristics inherent to the fact that, while judges were blind to experimental condition while making these ratings, participants were not. Unavoidably, when making judgments about the content of their own dreams, participants were aware of which task (AEMT or Control task) they had completed on that visit, and so may have been motivated to find connections.

### Memory reconsolidation in dreams?

According to reconsolidation theory, an already-consolidated remote memory can become destabilized when activated, potentially enabling it to be updated with new information [20, 53]. Sleep is well-documented to facilitate memory consolidation and may also be a state in which remote memory networks are *reconsolidated* after destabilization [5]. In line with a handful of earlier studies [25], our current observations suggest that new experiences can trigger dreams incorporating elements of related memories from the remote past. We speculate that this feature of dreaming may reflect the reconsolidation of remote memories during sleep, resulting from their activation and destabilization during earlier wakefulness.

### Limitations and future directions

We collected relatively few reports from REM sleep in this study, and the limited number of REM dreams may have prohibited us from detecting variation in the effect of the AEMT on dream content and emotion across sleep stage. As in much of our work, we focused here on NREM dreams because they are more likely than REM sleep dreams to incorporate episodic memory content [54], and are more practical to sample in quick succession. However, because REM dreams are on average more emotional than those recalled from NREM sleep, it could be that the AEMT would affect REM dreams differently from NREM dreams, especially in regard to emotion.

Another potential limitation of the current study was our selection of the control task. We chose a control writing prompt that was emotionally neutral and did not direct participants to recall an autobiographical memory. Given that this is the first study using this task, this was an intentional choice to maximize the difference between conditions, ensuring that if an effect existed, it could be clearly detected. A drawback of this stark contrast between conditions is that the control and experimental conditions differ in a variety of respects, including cognitive engagement, personal relevance, and emotional salience. Thus, we cannot conclude exactly which of these specific features of the AEMT prompt drove its effects on dreaming. Future research could address this by utilizing experimental conditions that separately manipulate emotional valence, emotional arousal, and personal relevance in a more fine-grained manner.

Due to the contrast between conditions, we expected that the control writing task would have little or no impact on dreaming. But in fact, participants perceived their dreams to be at least moderately related even to the control task. This may have prevented participant ratings from sensitively detecting the effect of the AEMT on dream content. Future studies might consider including a null control in which participants do not write about even an imagined scenario, in order to minimize the possibility of task-related dreams in the comparison condition.

The current study did not measure changes in memory following sleep and dreaming, and so is unable to provide direct evidence that dreaming is indeed associated with functions such as memory reconsolidation and integration. While we speculate that the appearance of remote memory fragments in dreams could signify that those memories are being modified, this speculation would require empirical testing in future studies. If dreaming about remote memory is indeed associated with reconsolidation, it should be possible to detect increased memory stabilization following dreaming, analogous to the manner in which dreams of recent experience are associated with improved memory performance for recently learned information [4]. This would be a fruitful direction for future research.

Finally, these observations could be useful in developing interventions to reduce unwanted affect associated with emotionally negative memories. Dreams have long been proposed to play an adaptive role in emotional regulation, with dreaming about an intensely emotional experience enabling the gradual reduction of emotional reactivity via a fear extinction process [55, 56]. Indeed, recent studies have had some success in reducing the negative affect associated with memories by experimentally reactivating them during sleep via the delivery of learning-related sensory cues (Targeted Memory Reactivation) [57–59]. Activating a negative autobiographical memory before sleep is a simple, alternative method that could similarly be leveraged to reduce negative affect associated with a memory.

## Conclusions

Dreams are not just a simple reverberation of recent experience. Together with other research, our current observations suggest that presleep experiences can trigger the activation of remote memory networks, resulting in dreams that incorporate elements of long-past experience, intermingled with details of other memories. This suggests a potential role for sleep and dreaming in not just strengthening recent memories, but also in restabilizing recently activated remote memory traces.

## Data Availability

All data and analysis code are publicly available on Open Science Framework, at https://osf.io/ewmp7/.
